# Supplementary material for: Experiences of Homeless Families in Parenthood: A Systematic Review and Synthesis of Qualitative Evidence
Source: Int J Environ Res Public Health. 2020 Apr 15;17(8):2712. doi: 10.3390/ijerph17082712 (PMC7215402; doi:10.3390/ijerph17082712)
Supplement: Supplementary file 1 [file ijerph-17-02712-s001.zip › Table S1, S2, S3 PDF/Table S1.pdf]

International Journal of
Environmental Research and Public Health

MDPI

Table S1. Final evaluation of the included studies (according to the method used by Lockwood) [1].

Quote	Q1	Q2	Q3	Q4	Q5	Q6	Q7	Q8	Q9	Q10
Anderson et al. 2006 [2]	Y	Y	Y	Y	Y	N	Y	Y	N	Y
Anthony et al., 2017 [3]	Y	Y	Y	Y	Y	N	Y	Y	Y	Y
Cosgrove & Flynn, 2005 [4]	N	Y	Y	NC	Y	N	Y	Y	N	NC
Haskett et al., 2018 [5]	Y	Y	Y	Y	Y	Y	Y	Y	Y	Y
Holtrop et al., 2015 [6]	Y	Y	Y	Y	Y	N	Y	Y	Y	NC
Lindsey, 1997 [7]	Y	Y	Y	Y	Y	N	N	Y	N	NC
Morris & Butt, 2003 [8]	N	Y	Y	Y	Y	N	Y	Y	Y	Y
Roche et al., 2018 [9]	Y	Y	Y	Y	Y	N	Y	Y	Y	Y
Sylvestre et al., 2018 [10]	Y	Y	Y	Y	Y	Y	Y	Y	Y	Y
Tischler et al., 2004 [11]	Y	Y	Y	NC	Y	N	Y	NC	Y	N
Vanleit et al., 2006 [12]	Y	Y	Y	Y	Y	N	Y	Y	Y	N
% Yes	81.82	100	100	81.82	100	18.18	90.91	90.91	72.73	54.55

N — No; NC — Not Conclusive; Y — Yes.

Q1. Is there congruence between the declared philosophical perspective and the research methodology? Q2. Is there consistency between the research methodology and the research's question(s)/objective(s)? Q3. Is there consistency between the research methodology and the data collection methods? Q4. Is there congruence between the research methodology and the data's representation and analysis? Q5. Is there congruence between the research methodology and the results' interpretation? Q6. Is there a statement that contextualises the researcher from a cultural or theoretical point of view? Q7. Are the researcher's influence in the research and the inverse phenomenon addressed? Q8. Are the participants and their opinions adequately represented? Q9. Is the research considered ethical, according to the current criteria, or, for recent studies, is there evidence of ethical approval by an appropriate committee? Q10. Do the conclusions drawn in the research report derive from the data's analysis/interpretation? **Note:** Q1, Q2, Q3, etc., refer to the questions included in the JBI Critical Appraisal Checklist for Qualitative Research [1].

References

1. Lockwood, C.; Porrit, K.; Munn, Z.; Rittenmeyer, L.; Salmond, S.; Bjerrum, M.; Loveday, H.; Carrier, J.; Stannard, D. Chapter 2: Systematic reviews of qualitative evidence. In *Joanna Briggs Institute Reviewer's Manual*; Aromataris, E., Munn, Z., Eds.; The Joanna Briggs Institute, 2017. Available online: https://reviewersmanual.joannabriggs.org/ (accessed on 30 January 2019).
2. Anderson, L.; Stuttaford, M.; Vostanis, P. A family support service for homeless children and parents: User and staff perspectives. *Child Fam. Soc. Work* **2006**, *11*, 119–127, doi:10.1111/j.1365-2206.2006.00399.x
3. Anthony, E.R.; Vincent, A.; Shin, Y. Parenting and child experiences in shelter: A qualitative study exploring the effect of homelessness on the parent-child relationship. *Child. Fam. Soc. Work* **2017**, *23*, 8–15, doi:10.1111/cfs.12376
4. Cosgrove, L.; Flynn, C. Marginalized mothers: Parenting without a home. *Anal. Soc. Issues Public Policy* **2005**, *5*, 127–143, doi:10.1111/j.1530-2415.2005.00059.x
5. Haskett, M.E.; Armstrong, J.; Neal, S.C.; Aldianto, K. Perceptions of triple P-positive parenting program seminars among parents experiencing homelessness. *J. Child Fam. Stud.* **2018**, *27*, 1957–1967, doi:10.1007/s10826-018-1016-510.1007/s10826-018-1016-5
6. Holtrop, K.; McNeil, S.; McWey, L.M. It's a struggle but I can do it. I'm doing it for me and my kids: The psychosocial characteristics and life experiences of at-risk homeless parents in transitional housing. *J. Marital Fam. Ther.* **2015**, *41*, 177–191, doi:10.1111/jmft.12050
7. Lindsey, E.W. The process of restabilization for mother-headed homeless families: How social workers can help. *J. Fam. Soc. Work* **1997**, *2*, 49–72, doi:10.1300/J039v02n03_05

8. Morris, R.I.; Butt, R.A. Parents' perspectives on homelessness and its effects on the educational development of their children. *J. Sch. Nurs.* **2003**, *19*, 43–50, doi:10.1177/10598405030190010701
9. Roche, S.; Barker, J.; McArthur, M. 'Performing' fathering while homeless: Utilising a critical social work perspective. *Br. J. Soc. Work* **2018**, *48*, 283–301, doi:10.1093/bjsw/bcx050
10. Sylvestre, J.; Kerman, N.; Polillo, A.; Lee, C.M.; Aubry, T.; Czechowski, K. A qualitative study of the pathways into and impacts of family homelessness. *J. Fam. Issues* **2018**, *39*, 2265–2285, doi:10.1177%2F0192513X17746709
11. Tischler, V.; Karim, K.; Rustall, S.; Gregory, P.; Vostanis, P. A family support service for homeless children and parents: Users' perspectives and characteristics. *Health Soc. Care Community* **2004**, *12*, 327–335, doi:10.1111/j.1365-2524.2004.00502.x
12. Vanleit, B.; Starrett, R.; Crowe, T.K. Occupational concerns of women who are homeless and have children: An occupational justice critique. *Occup. Ther. Heal. Care* **2006**, *20*, 47–62, doi:10.1080/J003v20n03_04

International Journal of
Environmental Research and Public Health
