# Supplementary material for: Experiences of Homeless Families in Parenthood: A Systematic Review and Synthesis of Qualitative Evidence
Source: Int J Environ Res Public Health. 2020 Apr 15;17(8):2712. doi: 10.3390/ijerph17082712 (PMC7215402; doi:10.3390/ijerph17082712)
Supplement: Supplementary file 1 [file ijerph-17-02712-s001.zip › Table S1, S2, S3 PDF/Table S2.pdf]

International Journal of
Environmental Research and Public Health

MDPI

Table S2. Summary of the conclusions reached through the CONQual tool (adapted from the one proposed in the Joanna Briggs Institute's Methodological Manual) [1].

Synthesised Finding	Type of Research	Dependability †	Credibility ‡	CONQual Score	Comments
The parents report: unsafe shelter environment; lack of hygiene; absence of privacy; limited space; isolated location, away from family, previous school and peers; changes in children's behaviour; and difficulties in children's education [2].	Qualitative			High	
The parents report an experience of disempowerment associated with shelter living (closely related to the rules, regulations and daily routines of the shelter). The children show confusion, sadness, anxiety/depression, abstinence, lack of appetite, development regressions, aggression and disrespect for authority [3].	Qualitative			High	
The parenthood experience was associated with the stigma of being a homeless mother. There was conflict, because the shelter's parental rules were not congruent with previous parenthood methods. There was also limited space for the family, and the shelter was located far from school. On the other hand, several coping mechanisms were identified, evidencing self-efficacy, perseverance, resilience and resistance [4].	Qualitative	Downgrade 1 level	Downgrade 1 level	Low	Downgrade of 1 level in dependability, due to the dependability of primary studies. Downgrade of 1 level in credibility, due to the integration of both unequivocal findings and merely credible findings.
The parents report living parental experiences in highly stressful environments. They emphasise the difficulties in establishing routines, due to the constant transition, as well as the difficulty in maintaining consistency with respect to parenthood, due to the involvement of other adults and the crowded living areas. They also report difficulties related to the shelter's restrictions [5].	Qualitative			High	

Table S2. Summary of the conclusions reached through the CONQual tool (adapted from the one proposed in the Joanna Briggs Institute's Methodological Manual) [1] (Cont.).

Synthesised Finding	Type of Research	Dependability †	Credibility ‡	CONQual Score	Comments
The shelter is perceived by the parents as an environment that emphasises the parental role, helping to form a sense of community and collective kinship. The parents recognise their parental responsibilities, and are able to maintain discipline and to identify their children's academic problems. On the other hand, the parents report: lack of privacy, separation from family members, imposition of external rules, and difficulty in raising and educating their children [6].	Qualitative		Downgrade 1 level	Moderate	Downgrade of 1 level in credibility, due to the integration of both unequivocal findings and merely credible findings.
The mothers mentioned that the shelter was disempowering, due to: external rules, lack of safety, separation of family members, and disregard for the parents' role. The mothers reported difficulty in responding to their children's needs, due to stress, also expressing difficulties in their interactions with the children [7].	Qualitative	Downgrade 1 level		Moderate	Downgrade of 1 level in dependability, due to the dependability of primary studies.
The parents' behaviour was characterized by the abdication of responsibility, as well as by the inability to assume a proactive posture regarding their children's education. The frequent mobility of the homeless families, combined with inadequate environments, the absence of a home address, and little or no communication, raised barriers to the children's education [8].	Qualitative		Downgrade 1 level	Moderate	Downgrade of 1 level in credibility, due to the integration of both unequivocal findings and merely credible findings.
The fathers describe a "failure to perform" that results in "detachment" from their children. The fathers also attempt to conceal their inability to comply with prescriptive social rules and the dominant representations of paternity [9].	Qualitative		Downgrade 1 level	Moderate	Downgrade of 1 level in credibility, due to the integration of both unequivocal findings and merely credible findings.
The parents report changes in their children's behaviour and difficulty in dealing with them. The shelters' environment was described by the families as restrictive, noisy, chaotic, and unsafe. They also reported: lack of hygiene; lack of space, preventing the use of the normal disciplinary strategies; and lack of privacy, enhancing isolation and routine alterations. Some participants mentioned the shelter's importance as a stabilising experience for the family, promoting family cohesion and providing social opportunities [10].	Qualitative			High	

Table S2. Summary of the conclusions reached through the CONQual tool (adapted from the one proposed in the Joanna Briggs Institute's Methodological Manual) [1] (Cont.).

Synthesised Finding	Type of Research	Dependability †	Credibility ‡	CONQual Score	Comments
The parents consider the shelter deficient, in terms of safety, hygiene, and privacy, being also located far from school. The parents claim that they need: help with childcare, information regarding school, and mental health counselling/support. Parental difficulties, associated with children's behavioural problems, were identified [11].	Qualitative	Downgrade 1 level	Downgrade 2 levels	Very Low	Downgrade of 1 level in dependability, due to the dependability of primary studies. Downgrade of 2 levels in credibility, due to the integration of both unequivocal findings and unsupported findings.
Parents report difficulties in parenting, including not being able to maintain family routines, and the lack of time for oneself and for one's children. Loneliness, insecurity and lack of privacy are felt. The separation from friends and family members is also referred [12].	Qualitative		Downgrade 1 level	Moderate	Downgrade of 1 level in credibility, due to the integration of both unequivocal findings and merely credible findings.

CONQual score attribution: All qualitative studies start with a "high" score; a downgrade of 1 level results in a "moderate" score; a downgrade of 2 levels corresponds to a "low" score; and a downgrade of 3 or more levels implies a "very low" score. † For detailed information about "dependability", please consult Table 4. ‡ Regarding "Credibility", when the results' synthesis only includes unambiguous findings (results which are accompanied by an illustration that is beyond reasonable doubt, being, therefore, not open to discussion), the original score remains unaltered. When all the findings included in the synthesis are equivocal (results which are accompanied by an illustration that does not present a clear association with them, being, therefore, open to discussion), the original score descends 2 levels. When the synthesis comprises a mixture of unambiguous and equivocal findings, the original score descends 1 level. When the synthesis comprises a mixture of equivocal and unsupported findings (results that are not supported by the collected data), the original score descends 3 levels. Finally, when the synthesis only includes findings which are not supported by the collected data, the original score descends 4 levels

References

1. Munn, Z.; Porritt, K.; Lockwood, C.; Aromataris, E.; Pearson, A. Establishing confidence in the output of qualitative research synthesis: The ConQual approach. *BMC Med. Res. Methodol.* **2014**, *14*, 1–7, doi:10.1186/1471-2288-14-108
2. Anderson, L.; Stuttaford, M.; Vostanis, P. A family support service for homeless children and parents: User and staff perspectives. *Child Fam. Soc. Work* **2006**, *11*, 119–127, doi:10.1111/j.1365-2206.2006.00399.x
3. Anthony, E.R.; Vincent, A.; Shin, Y. Parenting and child experiences in shelter: A qualitative study exploring the effect of homelessness on the parent-child relationship. *Child. Fam. Soc. Work* **2017**, *23*, 8–15, doi:10.1111/cfs.12376
4. Cosgrove, L.; Flynn, C. Marginalized mothers: Parenting without a home. *Anal. Soc. Issues Public Policy* **2005**, *5*, 127–143, doi:10.1111/j.1530-2415.2005.00059.x
5. Haskett, M.E.; Armstrong, J.; Neal, S.C.; Aldianto, K. Perceptions of triple P-positive parenting program seminars among parents experiencing homelessness. *J. Child Fam. Stud.* **2018**, *27*, 1957–1967, doi:10.1007/s10826-018-1016-510.1007/s10826-018-1016-5
6. Holtrop, K.; McNeil, S.; McWey, L.M. It's a struggle but I can do it. I'm doing it for me and my kids: The psychosocial characteristics and life experiences of at-risk homeless parents in transitional housing. *J. Marital Fam. Ther.* **2015**, *41*, 177–191, doi:10.1111/jmft.12050
7. Lindsey, E.W. The process of restabilization for mother-headed homeless families: How social workers can help. *J. Fam. Soc. Work* **1997**, *2*, 49–72, doi:10.1300/J039v02n03_05
8. Morris, R.I.; Butt, R.A. Parents' perspectives on homelessness and its effects on the educational development of their children. *J. Sch. Nurs.* **2003**, *19*, 43–50, doi:10.1177/10598405030190010701
9. Roche, S.; Barker, J.; McArthur, M. 'Performing' fathering while homeless: Utilising a critical social work perspective. *Br. J. Soc. Work* **2018**, *48*, 283–301, doi:10.1093/bjsw/bcx050
10. Sylvestre, J.; Kerman, N.; Polillo, A.; Lee, C.M.; Aubry, T.; Czechowski, K. A qualitative study of the pathways into and impacts of family homelessness. *J. Fam. Issues* **2018**, *39*, 2265–2285, doi:10.1177%2F0192513X17746709
11. Tischler, V.; Karim, K.; Rustall, S.; Gregory, P.; Vostanis, P. A family support service for homeless children and parents: Users' perspectives and characteristics. *Health Soc. Care Community* **2004**, *12*, 327–335, doi:10.1111/j.1365-2524.2004.00502.x
12. Vanleit, B.; Starrett, R.; Crowe, T.K. Occupational concerns of women who are homeless and have children: An occupational justice critique. *Occup. Ther. Heal. Care* **2006**, *20*, 47–62, doi:10.1080/J003v20n03_04
