# Supplementary material for: Experiences of Homeless Families in Parenthood: A Systematic Review and Synthesis of Qualitative Evidence
Source: Int J Environ Res Public Health. 2020 Apr 15;17(8):2712. doi: 10.3390/ijerph17082712 (PMC7215402; doi:10.3390/ijerph17082712)
Supplement: Supplementary file 1 [file ijerph-17-02712-s001.zip › Table S1, S2, S3 PDF/Table S3.pdf]

International Journal of
Environmental Research and Public Health

MDPI

Table S3. Characterization of the included studies.

Authors	Objective(s)	Scientific Field/Country	Type of study (design; data collection method)	Participants	Context	Authors' Conclusions
Lisa Anderson; Maria Stuttaford; Panos Vostanis [1]	To identify the parents' and the employees' perceptions of their experience with the family support team, and also to recognise ways of improving this service in the future.	Medicine (Psychiatry)/ United Kingdom	**Qualitative with participatory approach and reported assessment.** Multiple methods: in-depth interviews with service users; participatory learning events; written diaries; reflection activities; participant observation; and semi-structured interviews with families.	21 families (12 single-parent families led by women, 8 couples with children, and 1 single-parent family led by a man).	Temporary shelters, for homeless families.	The parents attributed importance to practical and therapeutic interventions during this period of family transition. However, there were many reported negative opinions about life in a temporary shelter environment, such as: insecurity; lack of hygiene; absence of privacy; limited space for the family; isolated location, away from family, previous school and peers. The parents also stress changes in the children's behaviour and difficulties in their education. Additionally, they mention practical necessities, and the need for support in pregnancy and in mental health promotion.
Elizabeth R. Anthony; Aviva Vincent; Yoonkyung Shin [2].	To explore the effect of homelessness on the parent-child relationship.	Social Sciences/ United States	**Qualitative.** Semi-structured interviews.	19 families (16 represented by women and 3 represented by men).	Transitional shelters, for homeless families.	The effects of homelessness on children that were reported by the parents included: confusion, sadness, anxiety/depression, abstinence, lack of appetite, development regressions, aggression, and disrespect for authority. The parents also mention their experience of disempowerment associated with living in the shelter, which is closely related to the rules, regulations, and daily life, of the latter. Both the parents and the children needed to spend some time apart, to relax, regroup, and recharge. The children also needed time to play with other children, without the presence of third parties.

Table S3. Characterization of the included studies (cont.).

Authors	Objective(s)	Scientific Field/Country	Type of study (design; data collection method)	Participants	Context	Authors' Conclusions
Lisa Cosgrove; Cheryl Flynn [3].	To identify the experiences of single mothers who live in shelters, and also to recognise their strengths and their coping skills. To gather the participants' recommendations concerning social policies and shelter programmes.	Psychology and Education/ United States	**Qualitative, with a participatory, interpretative and collaborative approach**. Interviews subjected to narrative analysis.	17 women.	Transitional shelters, for single-parent families led by homeless women.	The experience of homeless parenthood was associated with: the stigma of being a homeless mother; conflicts caused by dissimilarities between the shelter's parental rules and the previous parenthood options; limited space for the family; being far from school. On the other hand, coping mechanisms were identified in all the participants, with self-efficacy, perseverance, resilience, and resistance, being evidenced. The participants were also capable of recognising short-term and long-term objectives.
Mary E. Haskett; Jenna Armstrong; Sarah C. Neal; Kristen Aldianto [4].	To examine the opinions of parents living in shelters about a brief workshop on evidence-based parental education (the "Triple P — Positive Parenting Program").	Psychology/ United States	**Mixed (with qualitative and quantitative approaches).** Sequential explanatory model. Data analysis was descriptive and exploratory. Quantitative data was obtained using a parental satisfaction questionnaire, following the "Triple P" workshops. To further explore the findings, qualitative data was examined (derived from parents' focus groups assembled after three workshops).	Quantitative data collection: 284 families (238 represented by women). Qualitative data collection: 16 families (14 represented by women).	Transitional shelters, for homeless families.	The parents who live in shelters express strongly positive opinions concerning the workshops on parental education. With respect to the shelters, the parents report living parental experiences in highly stressful environments. They emphasise the difficulty in establishing routines, due to the constant transition, as well as the difficulty in maintaining parenthood consistency, due to the involvement of other adults and to the crowded living areas. They also mention problems related to the shelter's isolated location and to the shelter's restrictions.

Table S3. Characterization of the included studies (cont.).

Authors	Objective(s)	Scientific Field/Country	Type of study (design; data collection method)	Participants	Context	Authors' Conclusions
Kendal Holtrop; Sharde' McNeil; Lenore M. McWey [5]	To acquire a better understanding of the psychosocial status and life experiences of homeless parents who live in transitional housing.	Psychology (Family Therapy)/ United States	**Mixed (with qualitative and quantitative approaches).** Multi-method descriptive study. Quantitative data was obtained using a questionnaire on parental depression, parental stress, parental practices, and perception of child behaviour. Qualitative data was collected through semi-structured interviews.	Quantitative data collection: 69 parents/ primary caregivers (60 were women). Qualitative data collection: 24 parents/ primary caregivers (19 were women).	Transitional shelters, for homeless families.	On the one hand, parents see the transitional shelter as an opportunity that: highlights the parental role; helps to form a sense of community and collective kinship. On the other hand, they report: lack of privacy; separation from family members; imposition of external rules; and difficulty in raising and educating their children. The children are mentioned as a source of motivation, regarding the search for a better life and/or employment. They encourage the adoption of a positive and optimistic perspective, conducive to perseverance and hope for a better future. The parents recognise their parental responsibilities, and are able to maintain discipline and to identify their children's academic problems.
Elizabeth W. Lindsey [6].	To explore the process by which homeless families led by mothers are relocated.	Social Sciences/ United States	**Qualitative.** Semi-structured interviews. Methodology: grounded theory (including data analysis through constant comparison).	10 women.	Transitional shelters, for single-parent families led by homeless women.	The results point to a three-stage restabilisation process: Meeting the Family's Immediate Needs, Creating a New Home, and Maintaining Family Stability. The mothers described the shelter as: unsafe; separating family members; not promoting the parents' role; disempowering for the parents, due to external rules. Additionally, the mothers reported difficulty in responding to their children's needs, because of the experienced stress, also mentioning difficulties in their interactions with the children. For some women, their dependence on the shelter's services and resources became a problem.

Table S3. Characterization of the included studies (cont.).

Authors	Objective(s)	Scientific Field/Country	Type of study (design; data collection method)	Participants	Context	Authors' Conclusions
Rita I. Morris; Rachael A. Butt [7].	To explore the parents' perceptions of how homelessness has affected their children's development and academic performance.	**Nursing/** United States	**Qualitative.** Semi-structured interviews. Methodology: grounded theory.	34 families (28 represented by the mothers, 1 represented by the father, and 5 represented by relatives).	Multiple contexts (temporary shelters, motels, and transitional housing), for homeless families.	This study's results indicate that homeless children suffer from a lack of safety and parental attention, which contributes to their poor academic performance. The parents' behaviour was characterized by: abdication of responsibility; inability to assume a proactive posture regarding their children's education; blaming school authorities for the children's poor academic performance. The homeless families' frequent mobility, combined with inadequate environments, without a home address and with little or no communication, raised barriers to the children's education. In some adolescents, an ability to persevere, despite the absence of support, was identified. The children exhibited a great concern in hiding the fact that they lived in a shelter.
Steven Roche; Justin Barker; Morag McArthur [8].	To explore the meaning attributed by the fathers to their experiences of homelessness.	Social Sciences/ Australia	**Qualitative.** In-depth interviews and focus groups. Qualitative data analysis was performed using the NVivo software.	40 men.	Single-parent families led by homeless men. Recruited by agencies that provide support to the homeless.	The fathers describe a "failure to perform" that results in "detachment" from their children. The fathers also attempt to conceal their inability to comply with prescriptive social rules and the dominant representations of paternity.

Table S3. Characterization of the included studies (cont.).

Authors	Objective(s)	Scientific Field/Country	Type of study (design; data collection method)	Participants	Context	Authors' Conclusions
John Sylvestre; Nick Kerman; Alexia Polillo; Catherine M. Lee; Tim Aubry; Konrad Czechowski [9].	To explore the pathways and the perceived consequences of homelessness among families that live within an emergency family shelter system.	Psychology/ United States.	**Qualitative**. Semi-structured interviews, using a convenience sample.	18 families (represented by 14 women and 4 men).	Transitional shelters, for homeless families.	The shelters' environment was described by the families as restrictive, noisy, chaotic, and unsafe. They also reported: lack of hygiene; isolation; lack of space, preventing the use of the normal disciplinary strategies; lack of privacy; and routine alterations. The participants recognise changes in their children's behaviour, as well as difficulty in dealing with them. Feelings of judgment were identified and the need for mental health support was pointed out. Some participants mentioned the shelter's importance as: a way of accessing services and resources; a stabilising experience for the family; a promoter of family cohesion; a social opportunity.
Victoria Tischler; Khalid Karim; Sue Rustall; Peter Gregory; Panos Vostanis [10].	To identify the characteristics of homeless families referred to the family support service, and also to recognise the users' perspectives and experiences as temporary shelter residents.	Medicine (Psychiatry)/ United Kingdom	**Mixed (with qualitative and quantitative approaches).** Quantitative data was obtained using: the Hospital Anxiety and Depression Scale; the Parenting Daily Hassles Scale; the Eyberg Child Behaviour Inventory; and the Health of the Nation Outcome Scales for Children and Adolescents. Qualitative data was collected through semi-structured interviews.	49 families (33 single-parent families led by women, 14 couples with children, and 2 single-parent families led by men).	Temporary shelters, for homeless families.	High rates of parental difficulties, mental health problems, and related needs, were identified among the children and their parents. The parents' difficulties were associated with child behaviour problems. Needs connected with the access to existing community services (e.g. schools, health/social work visits) were also recognised. Parents consider the shelter to be lacking in safety, hygiene and privacy. Additionally, they mention needing: practical aid, help to deal with childcare issues, information about school education, and mental health counselling/support.

Table S3. Characterization of the included studies (cont.).

Authors	Objective(s)	Scientific Field/Country	Type of study (design; data collection method)	Participants	Context	Authors' Conclusions
Betsy VanLeit; Rebecca Starrett; Terry K. Crowe [11].	To describe the occupational objectives and the concerns of homeless mothers.	Occupational Therapy/ United States	**Qualitative and exploratory.** Semi-structured interviews.	27 women.	Transitional shelters, for single-parent families led by homeless women.	The most frequent occupational problems identified by the participants are related to: financing; employment; education; transportation; housing; time for oneself and for one's children; maintenance of family routines. The participants also report: feelings of loneliness; insecurity; separation from friends and family members; and lack of privacy.

References

1. Anderson, L.; Stuttaford, M.; Vostanis, P. A family support service for homeless children and parents: User and staff perspectives. *Child Fam. Soc. Work* **2006**, *11*, 119–127, doi:10.1111/j.1365-2206.2006.00399.x
2. Anthony, E.R.; Vincent, A.; Shin, Y. Parenting and child experiences in shelter: A qualitative study exploring the effect of homelessness on the parent-child relationship. *Child. Fam. Soc. Work* **2017**, *23*, 8–15, doi:10.1111/cfs.12376
3. Cosgrove, L.; Flynn, C. Marginalized mothers: Parenting without a home. *Anal. Soc. Issues Public Policy* **2005**, *5*, 127–143, doi:10.1111/j.1530-2415.2005.00059.x
4. Haskett, M.E.; Armstrong, J.; Neal, S.C.; Aldianto, K. Perceptions of triple P-positive parenting program seminars among parents experiencing homelessness. *J. Child Fam. Stud.* **2018**, *27*, 1957–1967, doi:10.1007/s10826-018-1016-510.1007/s10826-018-1016-5
5. Holtrop, K.; McNeil, S.; McWey, L.M. It's a struggle but I can do it. I'm doing it for me and my kids: The psychosocial characteristics and life experiences of at-risk homeless parents in transitional housing. *J. Marital Fam. Ther.* **2015**, *41*, 177–191, doi:10.1111/jmft.12050
6. Lindsey, E.W. The process of restabilization for mother-headed homeless families: How social workers can help. *J. Fam. Soc. Work* **1997**, *2*, 49–72, doi:10.1300/J039v02n03_05
7. Morris, R.I.; Butt, R.A. Parents' perspectives on homelessness and its effects on the educational development of their children. *J. Sch. Nurs.* **2003**, *19*, 43–50, doi:10.1177/10598405030190010701
8. Roche, S.; Barker, J.; McArthur, M. 'Performing' fathering while homeless: Utilising a critical social work perspective. *Br. J. Soc. Work* **2018**, *48*, 283–301, doi:10.1093/bjsw/bcx050
9. Sylvestre, J.; Kerman, N.; Polillo, A.; Lee, C.M.; Aubry, T.; Czechowski, K. A qualitative study of the pathways into and impacts of family homelessness. *J. Fam. Issues* **2018**, *39*, 2265–2285, doi:10.1177%2F0192513X17746709

10. Tischler, V.; Karim, K.; Rustall, S.; Gregory, P.; Vostanis, P. A family support service for homeless children and parents: Users' perspectives and characteristics. *Health Soc. Care Community* **2004**, *12*, 327–335, doi:10.1111/j.1365-2524.2004.00502.x
11. Vanleit, B.; Starrett, R.; Crowe, T.K. Occupational concerns of women who are homeless and have children: An occupational justice critique. *Occup. Ther. Heal. Care* **2006**, *20*, 47–62, doi:10.1080/J003v20n03_04
